# Investigating the relationship between right ventricular size and function with pre‐eclampsia: A two‐group cross‐sectional study

**DOI:** 10.1002/hsr2.1135

**Published:** 2023-02-28

**Authors:** Hedieh Alimi, Afsoon Fazlinejad, Maryam Emadzadeh, Milad Abouzari

**Affiliations:** ^1^ Vascular and Endovascular Surgery Research Center Mashhad University of Medical Sciences Mashhad Iran; ^2^ Department of Cardiology, Faculty of Medicine Mashhad University of Medical Sciences Mashhad Iran; ^3^ Clinical Research Development Unit, Ghaem Hospital Mashhad University of Medical Sciences Mashhad Iran; ^4^ Faculty of Medicine Mashhad University of Medical Sciences Mashhad Iran

**Keywords:** gestational hypertension, pre‐eclampsia, right ventricular function, strain

## Abstract

**Background and Aims:**

Pre‐eclampsia is a multisystem disorder characterized by symptoms of high blood pressure and proteinuria during pregnancy. It is associated with many complications and maternal and fetal mortality. This disorder may be associated with many cardiovascular complications and affect the function of the heart. Therefore, in this study, the structure and function of the right ventricle (RV) in patients with pre‐eclampsia have been investigated using echocardiography.

**Methods:**

This cross‐sectional study was conducted in Ghaem Hospital of Mashhad. Thirty‐two pregnant women, whose gestational age was 20 weeks or more, were considered as the case group after evaluating blood pressure and confirming proteinuria and pre‐eclampsia. Thirty‐two healthy pregnant women were also included in the study as a control group. The function of the RV was evaluated using two‐dimensional transthoracic echocardiography.

**Results:**

Investigating the results of the study shows that in pregnant women with pre‐eclampsia, RV fractional area change, and RV strain indices have decreased significantly compared with healthy pregnant women (*p* < 0.05). Also, the statistical analysis shows that no significant differences were observed in the two groups in terms of echocardiographic indices *E*, *A*, *É*, *E*/*É*, *É*/*Á*, *E*/*A*, pulmonary artery pressure, Tricuspid Annular Plane Systolic Excursion, right ventricular diameter, and left ventricle mass index.

**Conclusion:**

According to the results of the study, it can be generally said that pre‐eclampsia may be associated with changes in the function and echocardiographic indices of the RV and may result in cardiac complications.

## 1 INTRODUCTION

Today, the most important causes of death of pregnant mothers are infection, high blood pressure (pre‐eclampsia), and bleeding.^1^, ^2^ Pre‐eclampsia is the most common type of blood pressure disorder during pregnancy, which is defined as a progressive multisystem with systolic blood pressure higher than or equal to 140 mmHg (or diastolic blood pressure 90 mmHg) along with proteinuria after the 20th week of pregnancy.^3^ According to different studies, the prevalence of pre‐eclampsia varies in different countries (from 3.3% in Australia to 12% in Bangladesh), which has the highest rate of perinatal mortality associated with adverse pregnancy outcomes.^4^, ^5^ Pre‐eclampsia is the third cause of maternal mortality in the world and the second cause of maternal mortality in Iran.^6^ However, recent studies have shown that the prevalence of pre‐eclampsia is increasing in Iran.^4^

Despite various studies, the pathophysiology of pre‐eclampsia is not completely known.^7^ Some studies have stated that pre‐eclampsia is caused by maternal factors and fetal/placental factors. The incomplete connection between the placenta and the uterine wall and the lack of restoration of the spiral arteries of the decidua and myometrium in early pregnancy (weeks to months before the clinical manifestations of this disease) are known.^8^, ^9^ Lack of adequate placental blood flow leads to relative hypoxia in the trophoblast tissue, which can cause oxidative stress.^10^ This alters angiogenesis within the placental villi, leading to abnormal growth of fetal vessels and resulting in abnormal vascular changes. It seems that placental secretion of antiangiogenic factors (sFlt‐1 and endoglin) leads to maternal vascular dysfunction, hypertension, proteinuria, and other clinical symptoms of pre‐eclampsia.^11^, ^12^ In addition, changes in cardiac function caused by increased afterload of the heart as a result of increased vascular resistance lead to the development of disease.^13^ Studies have shown that focal hypertrophy caused by pre‐eclampsia causes a significant increase in end‐diastolic pressure, which leads to systolic and diastolic failure.^14^

Pre‐eclampsia is associated with various complications. Pre‐eclampsia and intrauterine growth restriction are associated with an increased risk of cardiovascular diseases for the mother in the future. Pre‐eclampsia, which is associated with insulin resistance, extensive endothelial damage and dysfunction, coagulation defects and increased systemic inflammatory response, increases the risk of cardiovascular diseases.^15^ Women with high blood pressure during pregnancy face an increased risk of cardiovascular diseases in the future. In particular, the history of pre‐eclampsia increases the risk of venous thromboembolic diseases and hemorrhagic stroke.^16^, ^17^ Considering the limitations of the studies conducted in this regard, the purpose of this study was to investigate the relationship between the size and function of the right ventricle (RV) and pre‐eclampsia.

## 2 METHODOLOGY

### 2.1 Ethical considerations

The procedures and objectives of the study were explained to the patient and informed consent was obtained. The participants' information was collected confidentially so as not to cause any concern for them. Finally, the information was analyzed and reported. The code of ethics was obtained from the ethics committee of Mashhad University of Medical Sciences under the number IR.MUMS.MEDICAL.REC.1397.133.

### 2.2 Study population and inclusion and exclusion criteria

In this case‐control study, 32 patients with pre‐eclampsia (after blood pressure assessment, proteinuria confirmation, and proof of pre‐eclampsia for the case group by the doctor based on ACOG guidelines^18^) were included as the case group and 32 pregnant women as the control group. Thirty‐two members of the control group were selected from among the volunteers who referred to Ghaem Hospital of Mashhad University of Medical Sciences from July 2017 to November 2018 by nonrandom and easy sampling method. The inclusion criteria of the case group included pregnant women with a gestational age of more than 20 weeks with pre‐eclampsia, sinus rhythm, and suitable echocardiographic image. Exclusion criteria were history of significant heart valve disease, congenital heart disease, history of heart surgery, history of pulmonary hypertension, history of smoking, atrial or ventricular arrhythmia, kidney diseases, lung diseases, liver diseases, history of hypertension before pregnancy, and diabetes. In the control group, healthy pregnant women with normal blood pressure and complete urine test and with a gestational age of more than 20 weeks were selected, and the people of this group were matched with the case group in terms of age and parity.

### 2.3 The sample size

The sample size of this study was 32 people in each group. This was obtained by using the average comparison formula of two independent communities. Considering *α* = 0.05, *β* = 0.2, and based on the study of Çağlar et al.^19^ and also considering RV basal diameter as an outcome, the sample size was calculated as 32 individuals in each group.

## 3 TOOLS AND DEVICES

Demographic information such as age, height, weight of patients, and medical records was collected in the checklist according to the objectives of the study. Also, echocardiography indices were measured by an echocardiography fellowship. To check different echocardiography indicators, a Siemens echocardiography machine, model ACUSON SC 2000, made in Germany, with a V1C4 probe and a frequency of 4.5–1.25 MHz was used. *E*/*E*′, right ventricular diameter (RVD), body surface area (BSA), RV fractional area change (FAC), Tricuspid Annular Plane Systolic Excursion (TAPSE), *E*, *A*, *E*/*A*, *E*′, *E*′/*A*′, pulmonary artery pressure (PAP), RV strain, and left ventricle (LV) mass index indices were recorded in the respective checklist. In the review of all indicators, the study of Lang et al.^20^ was used to define the indicators.

Myocardial strain measurement is a sensitive method to evaluate cardiac function. Myocardial strain is the percentage of shape change between two areas, such as the shortening of the myocardial muscle in systole or the increase in its length in diastole, which can be calculated in three directions: longitudinal, transverse, and radial. In this study, the basis of measuring RV strain in the longitudinal direction was in the free wall and interventricular septum.^21^

RVD is an indicator for measuring the diameter of the RV. The best way to measure RVD is in the RV‐focused apical four‐chamber view. In this study, the middle third of the RV (RV mid‐diameter) was measured at the end of diastole. Normal values are in the range of 1.9–3.5 cm, and values greater than 3.5 cm indicate right ventricular dilatation (Table 1).

**Table 1:** Comparison of demographic status between people with pre‐eclampsia and healthy pregnant people.

Status	People with pre‐eclampsia (*n* = 32)	Healthy pregnant people (*n* = 32)	Statistical test
	Mean ± SD	Mean ± SD	*p* Value
Age (year)	31.65 ± 6.05	30.75 ± 7.21	0.58**
BSA (kg/m^2^)	1.77 ± 0.2	1.73 ± 0.17	0.43**
Parity			
Nulliparity *N* (%)	12 (37.5)	17 (52.5)	0.2*
Multiparity *N* (%)	20 (62.5)	15 (46.9)	

Abbreviations: BSA, body surface area; SD, standard deviation.

* Chi‐squared.

** *t* Test.

FAC is an index to evaluate the overall systolic function of the RV and is measured by calculating the difference between the right ventricular surface area in systole and diastole divided by the right ventricular surface area in diastole. Values less than 35% are considered pathological.

TAPSE is an index to evaluate the right ventricular systolic function in the longitudinal direction, which is measured by the position adjusted along the lateral leaflet of the tricuspid valve in line with the lateral wall of the RV in the four‐chamber view. Values less than 17 mm indicate right ventricular systolic dysfunction.

Mean PAP in this study^20^ was calculated based on the following formula: 4 × (TR jet velocity)^2^ + right atrial pressure.

In this formula, right atrial pressure is defined based on inferior vena cava collapsibility. In addition, TR jet velocity is the regurgitation speed of the tricuspid valve, which represents the gradient difference between the RV and the right atrium.

Doppler flow through the tricuspid valve during diastole causes *E* and *A* waves. *E* wave is the speed of blood flow at the beginning of diastole and is created when the ventricle is actively filling. In addition, this wave depends on the pressure difference between the atrium and the ventricle and the compliance of the ventricle. *A* wave shows the speed of blood flow at the end of diastole and the time of atrial contraction. Tissue Doppler imaging technique can also be used to evaluate myocardial contraction and expansion. *é* wave appears at the beginning of diastole and *á* wave at the end of diastole.

Considering the way of measuring RV strain in which the septum is involved (global longitudinal RV strain) and due to the effect of pre‐eclampsia on the LV which increases the muscle mass of the LV,^19^ LV mass index was included in the study as a separate variable. The method of measuring LV mass in this study is based on the linear method and is as follows:

{(thickness of the interventricular septum + inner diameter of the LV + thickness of the lower lateral wall of the LV)3 − (inner diameter of the LV)3} + 0.6 g.

And finally, to get the index, the obtained number is divided by the BSA.

**Figure 1:**
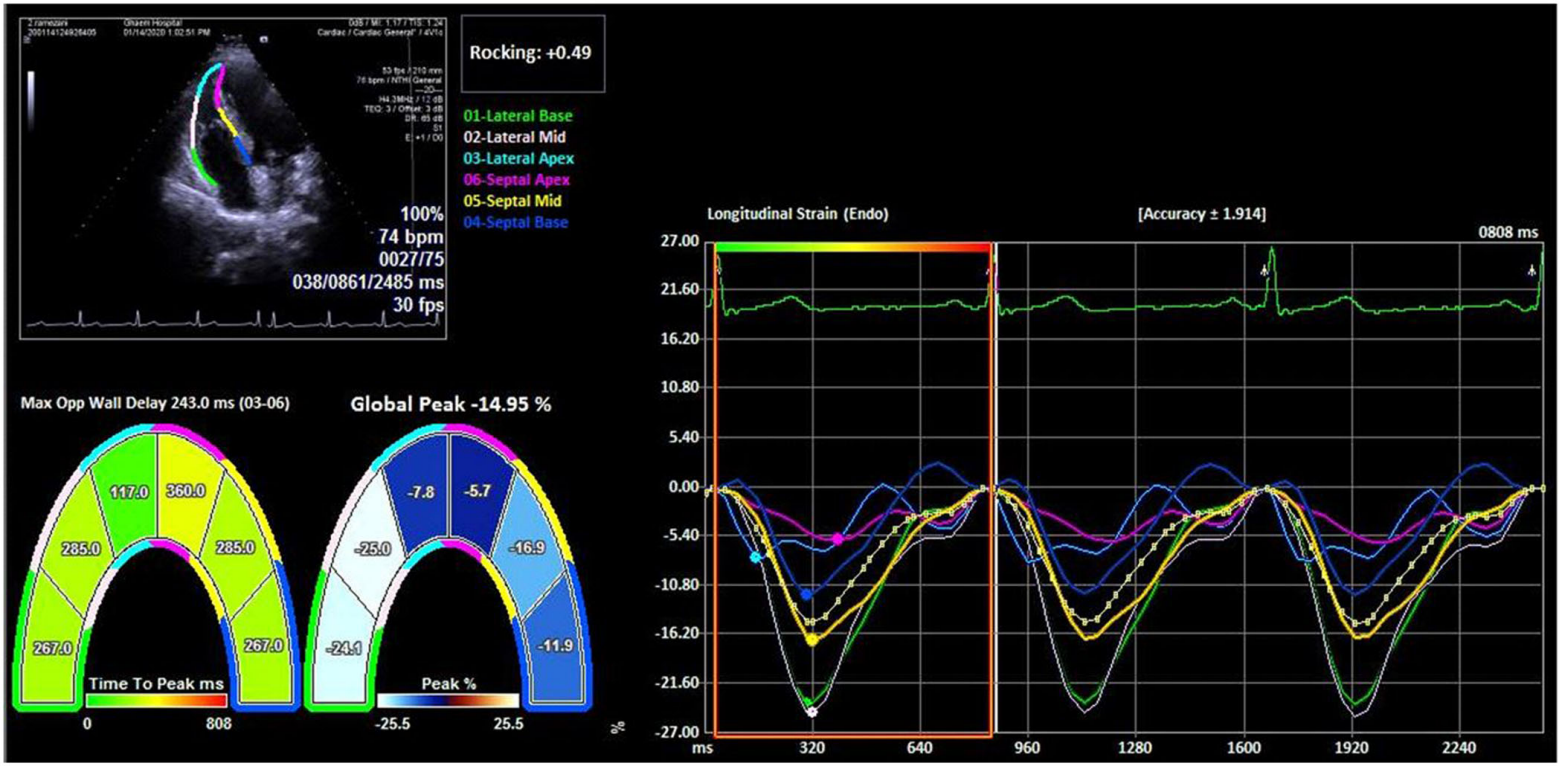
How to measure RV strain. RV, right ventricle.

The internal dimensions of the LV are best measured through the parasternal, perpendicular to the long axis of the LV and at the surface of the tips of the mitral valve leaflets. All measurements should be taken at the end of diastole (Figure 1).

## 4 DATA ANALYSIS

After collecting the data, the data were entered into SPSS software version 22. Mean and standard deviation were used to describe normally distributed quantitative data. Median and range (minimum–maximum) were reported for variables that were not normally distributed. To compare quantitative variables between groups, Mann–Whitney or *t* test was used. Chi‐squared test was used to compare qualitative variables with each other. The Spearman correlation test was used to indicate the correlation between variables. The significance level in all tests was considered less than 0.05. All reported *p* values are two‐sided.

## 5 RESULTS

In this study, the average age of the participants was 31.2 ± 6.6. There was no significant difference between the two groups in terms of age (*p* = 0.58). Among the participants, 29 people were nulliparity and 35 people were multiparity. The mean parity in these patients was 21.55 ± 0.5. In the investigation of the relationship between parity and pre‐eclampsia, it was shown that 37.5% of those with pre‐eclampsia was nulliparity and 62.5% of those with pre‐eclampsia was multiparity. The results of the statistical analysis do not show a significant difference (*p* = 0.2). Comparison of demographic status between people with pre‐eclampsia and healthy pregnant people is shown in Table 1.

The normal range of echocardiographic indicators on the right side of the heart is shown in Table 2.

**Table 2:** Normal range of echocardiographic indicators on the right side of the heart.

Indicator	Mean ± SD	Threshold of the abnormal range
RV FAC (%)	49 ± 7	<35
TAPSE (mm)	24 ± 5.3	<17
*E* (cm/s)	8/0 ± 7/5	<7
*A* (cm/s)	8/0 ± 4/4	<9/5
*E*/*A*	1.4 ± 0.3	<0.8 or >2
*E*′ (cm/s)	14 ± 1.3	<8.7
PAP (mmHg)	23 ± 5.2	>25
RV strain (%)	29 ± 5.4	>17
*E*/*E*′	4 ± 1	>6
RVD (cm)	2.7 ± 4	>3.5
	Normal range	
LV mass index (g/m^2^)	43–95	95<

Abbreviations: FAC, fractional area change; LV, left ventricle; PAP, pulmonary artery pressure; RV, right ventricle; RVD, right ventricular diameter; SD, standard deviation; TAPSE, Tricuspid Annular Plane Systolic Excursion.

Regarding our patients' echocardiographic indicators, mean, BSA, TAPSE, *E*, *A*, *E*/*A*, *E*′, PAP, RV strain, RV FAC, *E*/*E*′, RVD, and LV mass index are shown in Table 3.

**Table 3:** Mean echocardiographic indices among the participants.

Index	Mean ± SD
BSA (kg/m^2^)	1.75 ± 0.18
RV FAC (%)	38.40 ± 8.83
TAPSE (mm)	2.50 ± 1.98
*E* (m/s)	52.68 ± 17.31
*A* (m/s)	51.10 ± 14.48
*E*/*A*	1.0.5 ± 0.31
*E*′ (m/s)	11.74 ± 2.82
*E*′/*A*′	0.85 ± 0.33
PAP (mmHg)	22.84 ± 4.88
RV strain (%)	−19.95 ± 2.80
*E*/*E*′	4.65 ± 1.6
RVD (cm)	2.70 ± 0.39
LV mass index (g/m^2^)	57.14 ± 10.56

Abbreviations: BSA, body surface area; FAC, fractional area change; LV, left ventricle; PAP, pulmonary artery pressure; RV, right ventricle; RVD, right ventricular diameter; SD, standard deviation; TAPSE, Tricuspid Annular Plane Systolic Excursion.

The investigation of various echocardiographic indices in the population of pre‐eclampsia patients indicates that in this population, only in RV FAC indices, the number of abnormal people is higher than normal people. Table 4 shows the investigation of various echocardiographic indicators and its division into two categories, normal and abnormal, based on the normal range in this study.

**Table 4:** Distribution of echocardiographic indices among people with pre‐eclampsia.

Index	*N* (%)	
RV FAC (%)	Normal	17 (53.13)
	Abnormal	15 (46.87)
PAP (mmHg)	Normal	24 (75)
	Abnormal	8 (25)
*E*′/*A*′	Normal	20 (63.90)
	Abnormal	3 (37.9)
RVD (cm)	Normal	32 (100)
	Abnormal	0
TAPSE (mm)	Normal	31 (7.98)
	Abnormal	1 (3.1)
*E*	Normal	30 (75.93)
	Abnormal	2 (25.6)
*E*′	Normal	29 (90.63)
	Abnormal	3 (9.37)
*A*	Normal	23 (88.71)
	Abnormal	9 (12.28)
*E*/*E*′	Normal	27 (38.84)
	Abnormal	5 (6215)
LV mass index (g/m^2^)	Normal	29 (63.90)
	Abnormal	3 (37.9)
RV strain (%)	Normal	22 (75.68)
	Abnormal	10 (25.31)

Abbreviations: FAC, fractional area change; LV, left ventricle; PAP, pulmonary artery pressure; RV, right ventricle; RVD, right ventricular diameter; TAPSE, Tricuspid Annular Plane Systolic Excursion.

On the basis of the results of this study, RV FAC in healthy pregnant women was significantly higher than the group with pre‐eclampsia (*p* = 0.04); however, despite the significant difference, these changes are within the normal range. Other indicators were not significantly different between the group of healthy people and those with pre‐eclampsia (Table 5). The mean RV strain in the pre‐eclampsia group was −17.88 ± 2.35 and in the healthy group was −20 ± 2.71, which was a significant difference (*p* = 0.001). No significant difference was observed regarding TAPSE, *E*, *E*/*A* parameters between healthy and pre‐eclampsia groups (*p* > 0.05) (Table 5).

**Table 5:** Comparison of BSA, RV FAC, *A*, *E*′, *E*′/*A*′, PAP, *E*/*E*′, RVD, TAPSE, *E*, *E*/*A*, RV strain, and LV mass indices between people with pre‐eclampsia and healthy pregnant women.

Index	Healthy pregnant women (*n* = 32)	People with pre‐eclampsia (*n* = 32)	*p* Value
BSA (kg/m^2^)	1.73 ± 0.17	1.77 ± 0.2	0.43*
RV FAC (%)	38.40 ± 8.83	36.16 ± 9.11	0.04*
*A* (m/s)	48.03 ± 13.82	54.18 ± 14.69	0.08*
*E*′ (m/s)	11.71 ± 2.33	11.76 ± 3.28	0.94*
*E*′/*A*′	0.84 ± 0.37	0.85 ± 0.29	0.68*
PAP (mmHg)	22.15 ± 4.40	23.53 ± 5.29	0.26*
*E*/*E*′	4.57 ± 1.92	4.73 ± 1.22	0.69*
RVD (cm)	2.75 ± 0.40	2.64 ± 0.37	0.28*
TAPSE (mm)	2.25 ± 0.23	2.79 ± 2.75	0.75**
Median (min–max)	(0.47) [1.8–2.6] 2.3	(0.38) [1.6–18] 2.25	
*E* (m/s)	21.56 ± 52.40	12.01 ± 52.96	0.15**
Median (min–max)	(24.5) 43 [30–115]	(15) [32–90] 50	
*E*/*A*	0.35 ± 1.10	0.27 ± 1.01	0.37**
Median (min–max)	(0.44) [0.56–2.44] 1.00	[0.58–1.66] (0.49) 1.00	
RV strain (%)	2.71 ± − 20.30	2.35 ± − 17.88	<0.001**
median (min–max)	−20 [−16 to −27.9] (4.5)	−18 [−15 to −28] (2.66)	
LV mass index (g/m^2^)	56.4 ± 11.34	57.87 ± 9.83	0.54**
Median (min–max)	(40–77) 57	(37–80) 57.5	

*Note*: Means and standard deviations (SDs) were used for normally distributed data, and medians and ranges for data that are not normally distributed.

Abbreviations: BSA, body surface area; FAC, fractional area change; LV, left ventricle; PAP, pulmonary artery pressure; RV, right ventricle; RVD, right ventricular diameter; TAPSE, Tricuspid Annular Plane Systolic Excursion.

* *t* Test.

** Mann–Whitney test.

On the basis of the results of Table 6, a significant negative correlation was observed between the average RV FAC and RV strain (*p* < 0.001).

**Table 6:** Correlation of various echocardiographic indices and age with RV strain.

Index	RV strain	
	Correlation coefficient (*r*)	*p* Value
Age	−0.13	0.48
RV FAC	−0.644	<0.001
LV mass	−0.51	0.78
TAPSE	0.134	0.46
*E*	−0.11	0.54
*A*	−0.90	0.62
*E* · *A*	0.009	0.96
*É*	−0.239	0.19
PAP	−0.243	0.18
*E* · *É*	0.126	0.49
RVD	0.260	0.15

Abbreviations: FAC, fractional area change; LV, left ventricle; PAP, pulmonary artery pressure; RV, right ventricle; RVD, right ventricular diameter; TAPSE, Tricuspid Annular Plane Systolic Excursion.

## 6 DISCUSSION

According to the results of this study, the right ventricular function is impaired in women with pre‐eclampsia compared with healthy pregnant women. The changes of RV FAC variable in both groups are within the normal range. Due to the fact that the variable changes of RV strain are an indicator that can show right ventricular systolic dysfunction early in the subclinical stages, it can be used as a prognostic indicator to evaluate cardiac disorders after pre‐eclampsia. This raises the possibility that pre‐eclampsia as a multisystem disease, in addition to complications caused in pregnancy, can cause cardiac risks for mothers with pre‐eclampsia in the future.

This study was the first one conducted to accurately evaluate the right side of the heart in women with pre‐eclampsia in the Iranian female population. However, the number of studies evaluating echocardiography in women with pre‐eclampsia is increasing,^19^ however, most of the studies conducted in this regard have been conducted on left ventricular function.

In the study conducted by Çağlar et al.^19^ in 2015, 67 pregnant women with untreated pre‐eclampsia and 46 healthy pregnant women were examined. Right ventricular size and right ventricular outflow tract and free wall thickness, right atrium size at the end of systole were significantly higher in the pre‐eclampsia group than in the control group. Also, TAPSE index, isovolumic acceleration time, and myocardial function index were significantly lower in the pre‐eclampsia group compared with the control group. The results of this study show that pre‐eclampsia affects not only the left side of the heart but also the right side, which was in agreement with our study. Although in this study, the changes following pre‐eclampsia were reported in the normal range, but right ventricular dysfunction may be the cause of complications related to pre‐eclampsia. In our study, there was no difference in the size of the RV between the two groups, but the RV strain was significantly lower in the pre‐eclampsia group.

In the study conducted by Hassan et al.^22^ in 2019, it was stated that the changes in TAPSE, ESPAP, FAC, and right ventricular myocardial function index were significantly different between the two groups, which was consistent with our study. The results of this study show that echocardiography is an important evaluation method in examining women with pre‐eclampsia to prevent cardiac complications and mortality. Vaught et al.^23^ in 2018 stated that women with pre‐eclampsia had higher right ventricular systolic pressure levels, decreased right ventricular longitudinal strain (RVLSS), increased LA size, and increased LV wall thickness. Another important finding in this study was that 13% of women with pre‐eclampsia had grade 2 diastolic dysfunction. Also, this study confirms the reduction of RVLSS, which is probably due to RV dysfunction and increased right ventricular afterload, and it was consistent with our study.

## 7 LIMITATIONS

The main limitation of this research is the lack of investigation of the effect of gestational age and severity of pre‐eclampsia on the variables. In addition, measuring blood pressure before echocardiography and investigating the relationship of variables, especially RV strain with different levels of blood pressure can be helpful. The mentioned factors may affect the results of the study and cause different results.

## AUTHOR CONTRIBUTIONS

**Hedieh Alimi**: Conceptualization; data curation; formal analysis; investigation; methodology; project administration; resources; supervision; validation; visualization; writing—original draft; writing—review and editing. **Afsoon Fazlinejad**: Conceptualization; data curation; investigation; project administration; validation; visualization; writing—original draft; writing—review and editing. **Maryam Emadzadeh**: Conceptualization; data curation; formal analysis; investigation; methodology; project administration; supervision; validation; visualization; writing—original draft; writing—review and editing. **Milad Abouzari**: Conceptualization; data curation; formal analysis; investigation; methodology; project administration; supervision; validation; visualization; writing—original draft; writing—review and editing.

## CONFLICT OF INTEREST STATEMENT

The authors declare no conflict of interest.

## TRANSPARENCY STATEMENT

The lead author Milad Abouzari affirms that this manuscript is an honest, accurate, and transparent account of the study being reported; that no important aspects of the study have been omitted; and that any discrepancies from the study as planned (and, if relevant, registered) have been explained.

## Data Availability

The data that support the findings of this study are available from the corresponding author upon reasonable request. All authors have read and approved the final version of the manuscript. Milad Abouzari had full access to all of the data in this study and takes complete responsibility for the integrity of the data and the accuracy of the data analysis.
